# Timelygpt: extrapolatable transformer pre-training for long-term time-series forecasting in healthcare

**DOI:** 10.1007/s13755-025-00384-0

**Published:** 2025-10-14

**Authors:** Ziyang Song, Qincheng Lu, Hao Xu, Ziqi Yang, He Zhu, David Buckeridge, Yue Li

**Affiliations:** 1 School of Computer Science, McGill University, Montreal, Canada; 2 School of Population and Global Health, McGill University, Montreal, Canada; 3 Mila - Quebec AI Institute, Montreal, Canada; 4 School of Electrical Engineering and Computer Science, Ohio Universtiy, Athens, United States

**Keywords:** Time-series pre-training, Transfer learning, Biosignals, Irregularly-sampled time series, Forecasting, Classification

## Abstract

**Purpose:**

Large-scale pre-trained models (PTMs) such as BERT and GPT have recently achieved great success in Natural Language Processing and Computer Vision domains. However, the development of PTMs on healthcare time-series data is lagging behind. This underscores the limitations of the existing transformer-based architectures, particularly their scalability to handle large-scale time series and ability to capture long-term temporal dependencies.

**Methods:**

In this study, we present Timely Generative Pre-trained Transformer (TimelyGPT). TimelyGPT employs an extrapolatable position (xPos) embedding to encode trend and periodic patterns into time-series representations. It also integrates recurrent attention and temporal convolution modules to effectively capture global–local temporal dependencies.

**Results:**

Our experiments show that TimelyGPT excels in modeling continuously monitored biosignals and irregularly-sampled time series data commonly observed in longitudinal electronic health records (EHRs). In forecasting continuous biosignals, TimelyGPT achieves accurate extrapolation up to 6000 timesteps of body temperature during the sleep stage transition given a short look-up window (i.e., prompt) containing only 2000 timesteps. For irregularly-sampled time series, TimelyGPT with a proposed time-specific inference demonstrates high top recall scores in predicting future diagnoses using early diagnostic records, effectively handling irregular intervals between clinical records. We further demonstrate that TimelyGPT achieves strong discriminative performance on both continuous and irregularly-sampled time series.

**Conclusion:**

Together, we envision TimelyGPT to be useful in various health domains, including long-term patient health state forecasting, patient risk trajectory prediction, and disease classification. Its code is available at Github.

## Introduction

Time-series forecasting holds significant importance in healthcare, given its potential to trace patient health trajectories and predict medical diagnoses [1, 2]. In the field of healthcare, there are two primary categories: continuously monitored and irregularly-sampled time series data. Continuous time-series, such as biosignals, have been extensively studied in various applications, including health monitoring [3], disease classification [4], and physical activity prediction [5]. Irregularly-sampled time series are commonly found in clinical records, where spontaneous updates are made due to outpatient hospital visits or inpatient hospital stays [6]. The key challenge is to extract meaningful contextualized representations from the time-series to make accurate long-term forecasting. A promising approach is to adopt transfer learning [1]. Initially, a model is pre-trained on large-scale datasets to learn contextualized temporal representations. This pre-trained model (PTM) is then fine-tuned to forecast target sequences.

The recent impressive achievements of Transformer PTMs in Natural Language Processing (NLP) and Computer Vision (CV) domains have inspired growing interest in time-series Transformer-based PTMs. Time-Series Transformer (TST) uses a mask-and-reconstruction pre-training strategy to extract contextualized representations from time series [7]. Cross-Reconstruction Transformer (CRT) learns temporal representations by dropping and reconstructing certain segments from time series [8]. Additionally, Transformer PTMs have been applied to traffic [9], tabular [10], and speech time series [11].

Transfer learning by pre-training on large time-series data followed by fine-tuning for long-term time series forecasting (LTSF) tasks is a promising avenue. However, existing studies primarily focus on training from scratch on limited data for LTSF tasks [1]. These studies often introduce tailored architectures and attention modules to extract complex temporal dependencies [12–14]. However, the scalability of these transformers on large datasets for LTSF tasks remains an open question [15]. A recent study argues that the permutation-invariant nature of self-attention causes the loss of temporal information [16]. As a result, transformers often underperform compared to convolution-based models, potentially due to their struggles with local features and multi-scale features [17, 18]. Overall, existing research on time-series transformers often lacks rigorous evaluation on large datasets and does not consistently outperform conventional approaches on small data.

**Fig. 1:**
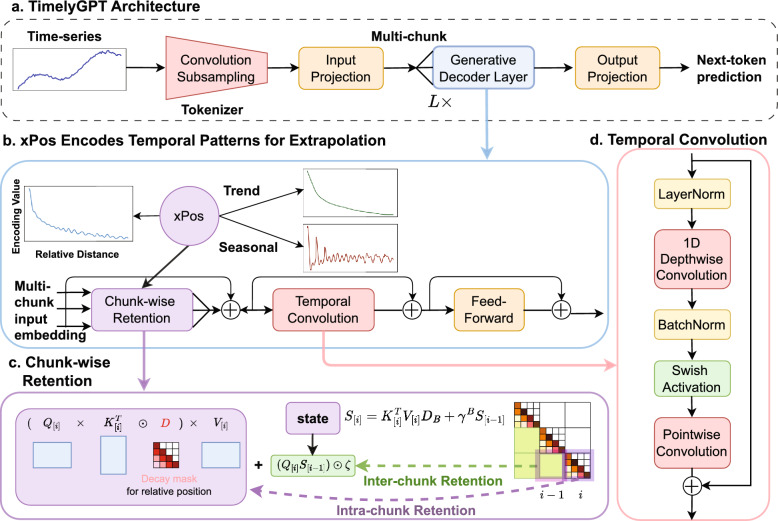
TimelyGPT overview. **a** TimelyGPT architecture consists of a convolution-subsampling tokenizer followed by *L* decoder layers. **b** Each decoder layer is coupled with extrapolatable position embedding (Sect. 3.2) that encodes trend and periodic patterns into time-series representations, facilitating forecasting with extrapolation ability. **c** Chunk-wise Retention consists of parallel intra-chunk Retention and recurrent inter-chunk Retention, effectively handling long sequences in continuously monitored biosignals (Sect. 3.3). **d** Temporal Convolution captures nuanced local interactions from time-series representations (Sect. 3.4)

In this study, we provide an in-depth analysis of existing time-series Transformer models, covering key aspects such as the attention mechanism and position embedding. We argue that the seeming inadequacy of current transformer-based models for time-series data is due to their inability to model large-scale time series. Once these challenges are resolved, we would observe the typical scaling law found in NLP and CV domains [15, 19]. Motivated by this insight, we present a novel framework called *Timely Generative Pre-trained Transformer* (TimelyGPT) (Fig. 1) that utilizes an extrapolatable position (xPos) embedding to encode trend and periodic patterns into time-series representations [20]. TimelyGPT integrates recurrent attention (also known as Retention) and convolution modules for effectively capturing both global temporal dependencies and nuanced local interactions [11, 21].

The key contributions of our research are threefold:

1. We employ extrapolatable xPos embedding (Fig. 1b) to encode both trend and periodic patterns into time-series representations, facilitating long-term forecasting;
2. We extend recurrent attention (Fig. 1c) to handle both continuous and irregularly-sampled time series data;
3. We introduce convolution subsampling tokenizer (Fig. 1a) to extract features from raw time-series and temporal convolution (Fig. 1d) to sift local features among the timesteps.

Overall, our experimental results reveal that TimelyGPT effectively extrapolates temporal representations for long-term forecasting. This leads to highly effective pre-training on large-scale time-series biosignals and longitudinal EHR data, and ultimately superior task-specific fine-tuning performance compared to the existing methods.

## Related work

### Self-attention in transformer

Transformer employs an encoder-decoder architecture composed of *L* layers of Transformer blocks [22]. Each block consists of a self-attention layer followed by a feed-forward layer. For an input embedding ${{\varvec{X}}}\in \mathbb {R}^{N \times d}$, where *N* is the number of tokens and *d* is the hidden size, the self-attention mechanism is defined as:

1 $$\begin{aligned} \text {Attention}({{\varvec{X}}}) = \text {Softmax}\left( \frac{{{\varvec{Q}}}{{\varvec{K}}}^\top }{\sqrt{d}} \right) {{\varvec{V}}}\end{aligned}$$

where ${{\varvec{Q}}}, {{\varvec{K}}}, {{\varvec{V}}}= {{\varvec{X}}}{{\varvec{W}}}_Q, {{\varvec{X}}}{{\varvec{W}}}_K, {{\varvec{X}}}{{\varvec{W}}}_V \in \mathbb {R}^{N \times d}$ are the Query, Key, and Value matrices, respectively. This self-attention allows Transformer to model long-term dependencies effectively, making it extensively utilized in NLP and CV domains.

Self-attention mechanism computes the dot product ${{\varvec{Q}}}{{\varvec{K}}}^\top$ prior to the softmax operation, resulting in a quadratic computational complexity of $\mathcal {O}(N^2 d)$. As sequence length increases, this quadratic complexity becomes bottleneck, making it challenging to train for longer sequences. Recent studies have explored attention-free modules such as Multi-Layer Perceptron (MLP) [23], implicit long convolution [24], and Recurrent Neural Network (RNN) [21, 25]. In particular, RNN-based attention modules have scaled up to 14 billion parameters while maintaining competitive performance with linear training and constant inference complexities. These modules are categorized as linear attention mechanisms, as they avoid quadratic complexity by replacing the softmax term with a kernelized approximation $\phi ({{\varvec{Q}}}_n)\phi ({{\varvec{K}}}_m^\top )$, where $\phi (\cdot )$ is nonlinear function [26]. Its output embedding is recast as an RNN:

2 $$\begin{aligned}&{{\varvec{O}}}_n = \frac{\phi ({{\varvec{Q}}}_n) \sum _{m=1}^N \phi ({{\varvec{K}}}_m^\top ) {{\varvec{V}}}_m}{\phi ({{\varvec{Q}}}_n) \sum _{m=1}^N \phi ({{\varvec{K}}}_m^\top )} \end{aligned}$$

Thus, the output embedding of the *n*-th token, ${{\varvec{O}}}_n$, depends on both $\sum _{m=1}^N \phi ({{\varvec{K}}}_m^\top ) {{\varvec{V}}}_m$ and $\sum _{m=1}^N \phi ({{\varvec{K}}}_m^\top )$, which are incrementally updated through cumulative sums and yield $\mathcal {O}(N)$ complexity. These modules are particularly well-suited for time-series modeling by effectively capturing sequential dependencies [27]. In this study, TimelyGPT integrates the Retention mechanism with the convolution modules to effectively capture both global and local contexts.

### Position embedding in transformer

Transformer relies on position embedding to capture temporal relations, since the self-attention mechanism alone does not inherently discern token order [28]. *Absolute* position embedding, which commonly employs sinusoidal functions, adds positional encoding directly to token embeddings. However, this method only encodes discrete position indexes, making it less effective for continuous timescales such as trend and periodic patterns in time-series data [16]. In contrast, speech transformers utilize *relative* position embedding to handle continuous time by encoding positional information relative to token distances [11]. Rotary Position Embedding (RoPE), prevalent in numerous large language models [29, 30], applies rotation matrices to encode information from relative distances [31]. Additionally, the RNN-based Transformer Receptance Weighted Key Value (RWKV) uses exponential decay to encode information based on relative distance [25]. Bridging these techniques, xPos embedding utilizes both rotation and exponential decay to effectively capture long-term dependencies [20].

One challenge for Transformer is *extrapolation*, i.e., forecasting sequences longer than those seen during training, due to the difficulty in generalizing position embeddings to unseen positions [32]. Encoder-decoder architectures often concatenate the input sequence with a zero-padded placeholder for the target sequence and predict all timesteps at once, while encoder-only models encode input sequence for forecasting [12, 33]. Both approaches struggle with extrapolation and rely heavily on their linear layer for forecasting [34], limiting their effectiveness in LTSF tasks. To address the issue, Attention with Linear Biases (ALiBi) adjusts attention with penalties linearly correlated with token distances [32]. Building on this, xPos embedding employs exponential decay to assign penalties based on relative distances [20]. Consequently, xPos can handle inference lengths up to eight times the training length while maintaining comparable performance. Our TimelyGPT extends xPos from the NLP domain to long-term forecasting in the time-series domain, focusing on exploring the underlying mechanisms that enable the temporal extrapolation.

## Methodology

### Overview of TimelyGPT

We consider two types of time-series data: continuous biosignals and irregularly-sampled medical records. For continuous biosignals, an input sequence *x* is denoted as $\{x_1, x_2, \ldots , x_T\}$ over *T* timesteps, with each timestep $x_t$ composed of *V* features. As illustrated in Fig. 1.a, TimelyGPT employs a convolution-subsampling tokenizer with two 1-D convolution layers, reducing the $T \times V$ down to an $N \times V$ sequence. These tokens are projected into an input embedding ${{\varvec{X}}}\in \mathbb {R}^{N \times d}$ with a projection layer. For irregularly-sampled medical records, a sequence is represented as $x = \{(x_1, t_1), \ldots , (x_N, t_N)\}$, where *N* denotes the number of samples. Each sample $(x_n, t_n)$ consists of an observation $x_n$ (e.g., a structured diagnostic code) and its associated timestamp $t_n$. Given the discrete nature of longitudinal EHR data, we replace the convolutional tokenizer with a learnable embedding codebook.

Our proposed TimelyGPT effectively pre-trains on unlabeled data using next-token prediction task to learn temporal representations (Fig. 1). It first processes time-series inputs using a convolution-subsampling tokenizer for token embedding (Fig. 1a). To extract meaningful temporal patterns, TimelyGPT integrates three technical contributions. First, TimelyGPT utilizes extrapolatable xPos embedding to encode trend and periodic patterns (Fig. 1b, Sect. 3.2). Second, TimelyGPT utilizes the Retention module to capture global content (Fig. 1c, Sect. 3.3). Third, TimelyGPT deploys the convolution module to capture the local content (Fig. 1d, Sect. 3.4). Integrating Retention and Convolution modules enables the modeling of interactions between global and local content.

During per-training, TimelyGPT utilizes a next-token prediction task to learn temporal representations from unlabeled data. Given a sequence with a [SOS] token, TimelyGPT predicts the subsequent tokens by shifting the sequence rightwards. At the last layer, each token’s output representation is fed into a linear layer for next-token prediction. The pre-training loss is mean squared error (MSE) for continuous signals (e.g., biosignal) or cross-entropy for discrete signals (e.g., diagnosis codes). For fine-tuning on downstream tasks, we use average pooled representations from the last layer.

### Extrapolatable position embedding encodes temporal patterns

As our first contribution, TimelyGPT employs xPos to encode relative positional information into token embeddings based on the distance $n-m$ between token *n* and *m* [20]. Given an input embedding ${{\varvec{X}}}\in \mathbb {R}^{N \times d}$ for *N* tokens at *d* embedding dimensions, xPos is integrated into the *n*-th token embedding ${{\varvec{X}}}_n$ through rotation matrix $e^{i \theta n}$ and exponential decay $\gamma ^n$:

3 $$\begin{aligned}&\tilde{{\varvec{Q}}}_n \tilde{{\varvec{K}}}_m = {{\varvec{X}}}_n {{\varvec{W}}}_Q (\gamma e^{i\theta })^{n-m} {{\varvec{X}}}_m {{\varvec{W}}}_K= \gamma ^{n-m} {{\varvec{Q}}}_n {{\varvec{K}}}_m\nonumber \\&\text {where}\quad {{\varvec{Q}}}_n = {{\varvec{X}}}_n {{\varvec{W}}}_{Q} e^{i\theta n}, \, {{\varvec{K}}}_m = {{\varvec{X}}}_m {{\varvec{W}}}_{K} e^{-i\theta m} \end{aligned}$$

where $\theta$ and $\gamma$ indicate position-dependent rotation and decay hyperparameters [20, 31]. The exponential decay $\gamma ^{n-m}$ determines the intensity of remembering historical information, while the rotation matrix $e^{i\theta n}$ captures the oscillation frequencies. This decay mechanism effectively attenuates the influence of distant tokens, aiding in capturing long-term dependencies and enhancing extrapolation ability [20].

While initially designed for language modeling, xPos provides a compelling way for time-series modeling, mirroring the seasonal-trend decomposition (Fig. 1c). Its exponential decay $\gamma ^{n-m}$ naturally concentrates on recent times while diminishing the influence of distant times, reflecting the trend momentum of time series. The rotation matrix $e^{i \theta (n-m)}$ captures the seasonal component of time series through sinusoidal oscillations. Thus, the decay $\gamma$ and the rotation $e^{i\theta }$ correspond to trend and periodic patterns, respectively.

In healthcare time-series, xPos embedding effectively encodes both trend and periodic patterns crucial for modeling continuous biosignals and irregular clinical records. For continuous biosignals, trend patterns such as body temperature and vital signs are key health indicators, while electrocardiograms (ECGs) exhibit periodic patterns reflecting the physiological rhythms of the human body. In irregularly-sampled clinical records, age-related susceptibility to illnesses is observed in longitudinal population studies using administrative health data [35, 36]. Some EHRs also exhibit periodic patterns, especially for chronic diseases like COPD, which have alternating exacerbation and recovery cycles.

We hypothesize that xPos embedding can encode these trend and periodic patterns into token embeddings. By harnessing xPos, TimelyGPT can effectively model long-term dependencies essential for time-series forecasting. In Sect. 6.2 and 6.3, we validated our hypothesis and explored the underlying mechanisms driving temporal extrapolation for forecasting beyond training length.

### Retention for continuous and irregularly-sampled time series

As our second contribution, we adapt the Retention mechanism to effectively handle continuous time-series data [21]. The Retention mechanism based on xPos can be reformulated as an RNN to naturally model time-series data. Given the xPos embedding in Eq 3, the forward-pass of the Retention mechanism can be computed in parallel over all tokens with a linear training complexity:

4 $$\begin{aligned}&{{\varvec{Q}}}_n = {{\varvec{X}}}_n {{\varvec{W}}}_{Q} e^{i\theta n}, \, {{\varvec{K}}}_m = {{\varvec{X}}}_m {{\varvec{W}}}_{K} e^{-i\theta m}, \, {{\varvec{V}}}= {{\varvec{X}}}{{\varvec{W}}}_V \nonumber \\&\text {Ret}({{\varvec{X}}}) = ({{\varvec{Q}}}{{\varvec{K}}}^\top \odot {{\varvec{D}}}) {{\varvec{V}}}, \, {{\varvec{D}}}_{nm} = {\left\{ \begin{array}{ll} \gamma ^{n-m}, & n \ge m\\ 0, & n < m \end{array}\right. } \end{aligned}$$

where the decay matrix ${{\varvec{D}}}\in \mathbb {R}^{N \times N}$ and rotation matrix $e^{i\theta (n-m)}$ encode trend and periodic patterns into token embedding, taking into account the distance between tokens $n-m$. We use a multi-head Retention design where each head is assigned a distinct decay rate $\gamma _h = 1-2^{-5-h}$, with *h* as the head index [21]. This allows each head to learn different forgetting patterns and capture a range of long-term temporal dependencies. When reformulated as an RNN, the Retention in Eq. 4 can be manifested in a recurrent forward-pass with constant inference complexity by introducing a state variable ${{\varvec{S}}}_n \in \mathbb {R}^{d \times d}$:

5 $$\begin{aligned} \text {Ret} ({{\varvec{X}}}_n) = {{\varvec{Q}}}_n {{\varvec{S}}}_n , \, {{\varvec{S}}}_n = \gamma {{\varvec{S}}}_{n-1} + {{\varvec{K}}}_n^\top {{\varvec{V}}}_n \end{aligned}$$

This reformulated RNN excels in capturing sequential dependencies from the time-series. To handle long sequences, we use chunk-wise Retention by segmenting the sequence into multiple, non-overlapping chunks (Fig. 1c). We follow prior work in setting the chunk size to 512 [21]. Consequently, chunk-wise Retention maintains a linear complexity for long sequences.

To handle irregularly-sampled time series, we adapt the parallel Retention (Eq. 4) by explicitly accounting for varying intervals between observations. Given two samples $s_n$ and $s_m$, the decay mask ${{\varvec{D}}}$ is adapted according to the time gap $\Delta t_{n, m} = t_n - t_m$:

6 $$\begin{aligned} \text {Ret}({{\varvec{X}}}) = ({{\varvec{Q}}}{{\varvec{K}}}^\top \odot {{\varvec{D}}}) {{\varvec{V}}}, \, {{\varvec{D}}}_{nm} = {\left\{ \begin{array}{ll} \gamma ^{\Delta t_{n,m}}, & n \ge m \\ 0, & n < m \end{array}\right. } \end{aligned}$$

**Fig. 2:**
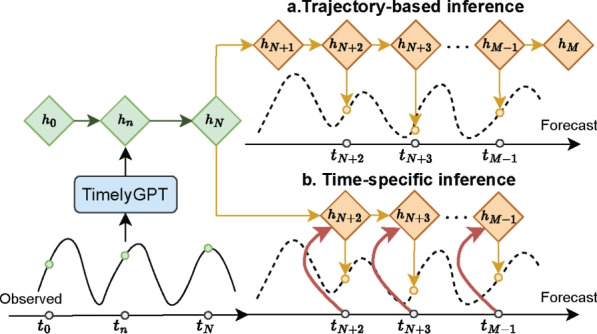
Two inference strategies for forecasting irregularly-sampled time series. (a) Trajectory-based inference. TimelyGPT autoregressively predicts the entire sequence at equal time gaps and selects the target data point from the inferred trajectory. (b) Time-specific inference. TimelyGPT directly predicts the target data point using historical hidden states and the gap between the target timestep and the last observed timestep

We also adapt the recurrent form in Eq. 5 for irregularly-sampled data by integrating time gaps into the update of the recurrent state variable:

7 $$\begin{aligned} \text {Ret}({{\varvec{X}}}_{n}) = {{\varvec{Q}}}_{n} {{\varvec{S}}}_{n}, \, {{\varvec{S}}}_{n} = \gamma ^{\Delta {t}_{n,n-1}} {{\varvec{S}}}_{n-1} + {{\varvec{K}}}_{n}^\top {{\varvec{V}}}_{n} \end{aligned}$$

where the base case ${{\varvec{S}}}_1=\textbf{0}$ in this recurrent update rule.

At inference time, to forecast irregularly-sampled time series, we consider two recurrent inference strategies, namely trajectory-based and time-specific inference (Fig. 2). The former autoregressively predicts a trajectory at equal time intervals. The latter directly makes prediction at a specific time point $s_{n'} = (x_{n'}, t_{n'})$. Specifically, knowing the target timestep $t_{n'}$ and the last observed sample $s_{n} = (x_{n}, t_{n})$, TimelyGPT outputs the embedding of the target token $\text {Ret}(X_{n'})={{\varvec{Q}}}_{n'}{{\varvec{S}}}_{n'}$, taking into account the time gap $\Delta t_{n', n} = t_{n'} - t_{n}$ and the recurrent state then becomes ${{\varvec{S}}}_{n'} = \gamma ^{\Delta {t}_{n',n}} {{\varvec{S}}}_{n} + {{\varvec{K}}}_{n}^\top {{\varvec{V}}}_{n}$.

### Convolution modules for local interaction

Convolution methods excel at detecting localized interactions from time series [37]. As the first part of our third contribution, we propose a **convolution-subsampling tokenizer** for feature extraction from the raw time-series input (Fig. 1a). Briefly, it uses multiple 1-D convolution layers to condense the time dimension and extract local features of the time-series. The convolution-subsampling tokenizer consists of two 1-D convolution layers with kernel size 3 and stride 2, reducing the sequence length to 1/4. Unlike the prevalent patching technique, which merely segments adjacent timesteps and features [33], the convolution tokenizer effectively captures local temporal interactions.

As the second part of our third contribution, we propose a *temporal convolution module* using a depth-wise separable convolution [38], sifting local temporal features from the time-series representations. As shown in Fig. 1d, this module starts with a layer normalization, followed by a 1-D depth-wise convolution and a point-wise convolution layer, with batch normalization and swish activation after the depth-wise convolution. Integrating convolution and attention allows TimelyGPT to extract global–local feature interactions [11, 39]. By stacking multiple decoder layers, each with a convolution layer, TimelyGPT discerns multi-scale features that characterize patterns across varying time scales [18].

### Computational complexity

TimelyGPT with its efficient Retention mechanism achieves $\mathcal {O}(N)$ training complexity and $\mathcal {O}(1)$ inference complexity. In contrast, BERT and GPT incur $\mathcal {O}(N^2)$ training complexity and $\mathcal {O}(N)$ inference complexity [26]. The vanilla attention mechanism in the Transformer, $\text {Attention}({{\varvec{X}}}) =\text {Softmax} (\frac{{{\varvec{Q}}}{{\varvec{K}}}^\top }{\sqrt{d}}) {{\varvec{V}}}$, introduces a training complexity of $\mathcal {O}(N^2d)$. This quadratic computational bottleneck prevents standard Transformer models from modeling long sequences (i.e., $N>> d$).

TimelyGPT achieves linear training complexity by following research in linear transformers [26]. In the Retention mechanism, $\text {Ret}({{\varvec{X}}}_n) = {{\varvec{Q}}}_n {{\varvec{S}}}_n, {{\varvec{S}}}_n= {{\varvec{K}}}_n^\top {{\varvec{V}}}_n + \gamma {{\varvec{S}}}_{n-1}$, both ${{\varvec{Q}}}_n {{\varvec{S}}}_n$ and ${{\varvec{K}}}_n^\top {{\varvec{V}}}_n$ have $\mathcal {O}(d^2)$ complexity. By recursively updating over *N* timesteps, the total complexity becomes $\mathcal {O}(N d^2)$. For inference, TimelyGPT proposes time-specific and trajectory-based methods. The trajectory-based inference recursively generates sequences with equally-spaced time intervals like the GPT model, incurring $\mathcal {O}(N)$ inference complexity. In contrast, the time-specific inference directly predicts target time point with $\mathcal {O}(1)$ complexity. Therefore, TimelyGPT achieves $\mathcal {O}(N)$ training complexity and $\mathcal {O}(1)$ inference complexity, making it computationally efficient and suitable for long sequences.

## Datasets

For continuous time-series, we used three publicly available, large-scale datasets for pre-training: (1) the Sleep-EDF dataset with 7 types of biosignals across 1.2 billion timesteps [40]; (2) the PTB-XL dataset with 12 variates of electrocardiogram data totaling 109 million timesteps [41]; (3) the PPG-Dalia dataset with 4 variates of photoplethysmograph data from 16.6 million timesteps [42]. Additionally, we employed six other datasets for downstream discriminative tasks, namely Epilepsy [43], UK Biobank (UKB) [44], EMG [45], RR, HR, SpO2 [46]. For irregularly-sampled time series, we utilized a large-scale Population Health Record (PopHR) database, which was established to monitor population health in Montreal, Quebec, Canada [47, 48]. The administrative data consist of the medical histories of 1.2 million patients in the form of International Classification of Diseases (ICD) codes. Specifically, we converted ICD-9 codes to coarse-grained phenotype codes (PheCodes) using the expert-defined PheWAS [49, 50]. We provide dataset description in Appendix A.1, and pre-processing in Appendix A.2.

## Experiments

We first validated the scaling pattern of TimelyGPT, determining the optimal number of model parameters for different dataset sizes (Sect. 6.1). We then explored TimelyGPT ’s extrapolation capabilities for long-term forecasting up to 6000 timesteps in Sleep-EDF’s biosignal data, and analyzed extrapolation’s underlying mechanism through visualization (Sect. 6.2). Our evaluation extended forecasting to irregularly-sampled time series (Sect. 6.3). Furthermore, we conducted ablation studies to evaluate the contributions of various components (Sect. 6.6).

### Pre-training and fine-tuning

During pre-training, TimelyGPT utilizes a next-token prediction task to learn general temporal representations from unlabeled data [51]. Given a sequence with a [SOS] token, TimelyGPT predicts the subsequent tokens by shifting the sequence to the right. At the last layer, each token’s output representation is fed into a linear layer for next-token prediction. The pre-training loss is Mean Squared Error (MSE) for continuous signals (e.g., biosignal) and cross-entropy for discrete signals (e.g., diagnosis codes).

Among other Transformer baselines, PatchTST adopted a masking-based approach, masking 40% of its patches as zeros [33]. CRT utilized a dropping-based pre-training, discarding up to 70% of patches [8]. For the Transformer models without established pre-training methods, we used a masking-based method by randomly masking 40% of timesteps [7]. For downstream forecasting tasks, we employed end-to-end fine-tuning on the entire model. The final linear layer was utilized for making the forecasts. All Transformer models performed 20 epochs of pre-training with MSE loss, followed by 5 epochs of end-to-end fine-tuning.

### Jointly forecasting multivariate biosignals from Sleep-EDF dataset

We utilized all seven variables from the Sleep-EDF dataset for a multivariate forecasting task, applying standardization as preprocessing. The Sleep-EDF dataset was split into training (80%), validation (10%), and test (10%) sets. All models were pre-trained on the entire training set and fine-tuned on a randomly chosen 20% subset of the training data, with time-series data segmented into non-overlapping sequences. For pre-training, we chose an input length of 4000 timesteps. For fine-tuning, we used a look-up window of 2000 timesteps and varied forecasting windows of 720, 2000, and 6000 timesteps. We used MAE as a metric. We evaluated TimelyGPT against Informer [12], Autoformer [13], FEDformer [14], PatchTST [33], TimesNet [52], TS2Vec [17], and DLinear [16].

### Forecasting irregularly-sampled diagnostic codes from PopHR dataset

We assessed long-term forecasting task of the irregularly-sampled time series extracted from the PopHR database. We divided the dataset into training (80%), validation (10%), and testing (10%) sets. We pre-trained on the entire training set and fine-tuned on a 20% subset of training data. We used cross entropy and top-*K* recall to evaluate the pre-training and fine-tuning, respectively. For forecasting, we set the look-up window to be 50 timestamps and the rest as the forecasting window, containing up to more than 100 timestamps (i.e., diagnosis codes). For our TimelyGPT, we separately evaluated the performance of trajectory-based and time-specific inferences (Sect. 3.3). We compared with several transformer baselines, including Informer, Fedformer, AutoFormer, and PatchTST as well as the models designed for irregularly-sampled time series, namely mTAND [53] and SeFT [54]. Given that diagnoses are discrete values, there was no need to utilize the convolution-subsampling tokenizer for TimelyGPT. Furthermore, we specified a patch size of 2 for PatchTST, indicating that every two timestamps are projected into a single patch.

### Discriminative tasks for continuous biosignals

We focus on discriminative tasks, such as classification and regression, across continuous biosignal datasets. To demonstrate the discriminative potential of the learned embeddings, we qualitatively evaluated ECG embedding using the UK Biobank dataset, revealing distinct clusters aligned with clinical disease labels. The UKB is a large-scale cohort comprising approximately 500,000 participants aged 40 to 69 at recruitment [44]. We used 46,321 resting-state clinical 12-lead ECG recordings from this dataset, each containing 5000 timesteps. We extracted eight disease labels from cardiologist reports using a published rule-based pipeline [55].

We then quantitatively evaluated the model on both classification and regression tasks using continuous biosignal datasets. For classification, we pre-trained on the Sleep-EDF [40] and PTB-XL datasets [41] separately. Subsequently, the PTMs were fine-tuned on the Sleep-EDF, Epilepsy [43], PTB-XL, and EMG [45] datasets. For regression, we pre-trained on both PTB-XL and PPGDalia datasets [42], followed by fine-tuning on the IEEEPPG [56], RR, HR, and SpO2 [46] datasets. Each dataset was split into a training (80%), validation (10%), and test (10%) sets. When both pre-training and fine-tuning on the same dataset, we fine-tuned the PTMs using 20% of the training set. We used accuracy and MAE as evaluation metrics for classification and regression, respectively. We assessed various transformer baselines, including PatchTST [33], AutoFormer [13], FedFormer [14], TimesNet [52], TST [7], and CRT [8] as well as consistency-based PTMs including TS-TCC [2], and TS2Vec [17].

**Fig. 3:**
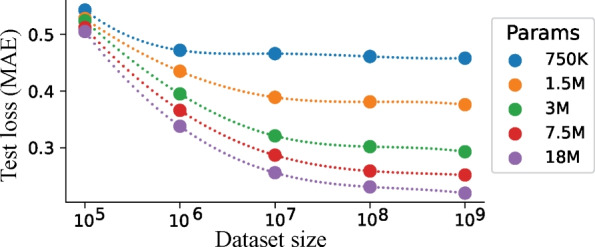
Test MAE of forecasting Sleep-EDF biosignals as a function of dataset sizes and parameter sizes. Both look-up and forecasting windows were set to 256 timesteps. TimelyGPT with more parameters tends to exhibit better performance when trained on larger datasets

**Table 1:** Comparison of TimelyGPT as well as 7 baselines for long-term forecasting experiment on the large-scale SleepEDF dataset

Metrics	MAE		
Window Size	720	2000	6000
TimelyGPT	0.542	**0.567**	**0.575**
Informer	0.675	1.013	1.256
Autoformer	0.532	0.908	1.026
Fedformer	0.515	0.865	0.912
PatchTST	**0.456**	0.768	*0.824*
DLinear	0.521	0.840	0.929
TS2Vec	0.602	1.231	1.204
TimesNet	*0.471*	*0.742*	0.865

Bold and italic numbers indicate the best and second best results for each metric and window

**Fig. 4:**
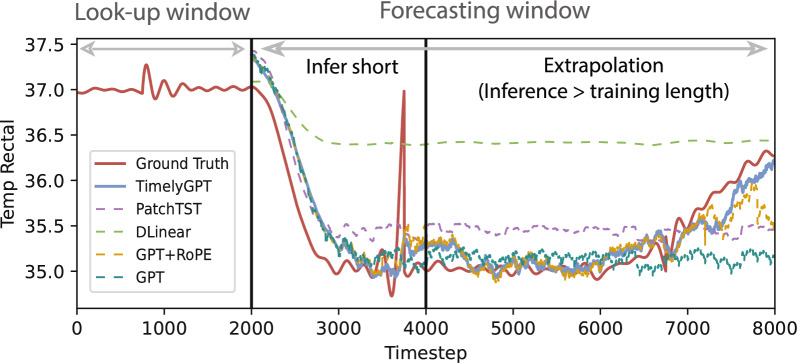
Predicted biosignal sequence of 6000 timesteps. Given a 2000 look-up window, we applied TimelyGPT (blue solid line) and 4 baseline methods (dashed lines) to predict the next 6000 timesteps. The groundtruth biosignals are displayed as red solid line. The two vertical lines demarcate the look-up window and the length of pre-training sequences, respectively

### Multi-label classification on irregularly-sampled time series

PopHR provides rule-based labels for 12 phenotypes, facilitating multi-label classification. For each disease label, we calculated the prevalence ratio of each PheCode across the true patients and the patient population, selecting the PheCodes with a ratio above 5. We excluded patients with fewer than 10 PheCodes, yielding a dataset comprising 47,000 patients and 8.4 million records. We used cross entropy and Area under Precision Recall Curve (AUPRC) to evaluate pre-training and fine-tuning, respectively. We compared TimelyGPT with the PTMs, which have demonstrated efficiency in Sect. 6.4, including PatchTST, TST, CRT, TS-TCC, and TST. We also assessed algorithms designed for irregularly-sampled time series, including mTAND [53], GRU-D [57], SeFT [54], and RAINDROP [6].

### Model parameters

For all benchmark experiments, we tailored the architecture and parameters of TimelyGPT based on the scaling-law analysis (Sect. 6.1; Fig. 3). Specifically, for the Sleep-EDF dataset, TimelyGPT was configured with 18 million parameters, and for the PopHR dataset, it was configured with 7.5 million parameters. While different Transformer models may have unique optimal hyperparameters, optimizing each model’s setup is computationally prohibitive with our current compute resources. For fairness of comparison, we compared TimelyGPT against all Transformer baselines at the same model size (Table 7). All experiments were conducted using an NVIDIA A100 GPU with 40 GB of memory. We also summarize the training time per epoch for TimelyGPT and baselines on the Sleep-EDF and PopHR datasets in Table 8.

## Results

### Scalability of TimelyGPT

We evaluated the scalability of TimelyGPT on the large-scale Sleep-EDF dataset to determine the optimal model parameters with respect to different dataset sizes [40]. We selected subsets of the Sleep-EDF dataset with timesteps ranging from $10^5$ to $10^9$, splitting each dataset into training (80%), validation (10%), and testing (10%) sets. Both look-up and forecasting windows were set to 256 timesteps for this experiment. TimelyGPT ’s performance improves as parameter and dataset size increase (Fig. 3), which is attributed to its capacity to handle more data, known as the scaling law for Transformer [15].

### Forecasting multivariate Sleep-EDF biosignals

TimelyGPT achieved the best performance in forecasting biosignals for all windows in terms of MAE, except for window 720 (Table 1). PatchTST achieved the best MAE at 0.456, whereas TimelyGPT conferred comparable performance. DLinear was also effective for the 720-timestep forecasting window. However, as the forecasting window increased to 2000 and 6000 timesteps, both PatchTST and DLinear suffered performance drops due to their reliance on the linear layers and inability to extrapolate beyond the training length. In contrast, pre-trained on 4000 timesteps, TimelyGPT consistently maintained superior performance up to 6000 timesteps given a short look-up window (i.e., prompt) containing only 2000 timesteps. These results underscore TimelyGPT ’s extrapolation capabilities in long-term forecasting, aligning with the findings in the NLP domain [20].

**Table 2:** Forecasting results of TimelyGPT and baselines on PopHR’s irregular-sampled time series dataset

Metrics	Recall @$K$ (%)		
$K$	5	10	15
TimelyGPT (trajectory-based)	52.30	64.35	77.12
TimelyGPT (time-specific)	**58.65**	**70.83**	*82.69*
Informer	46.37	60.14	71.24
Autoformer	42.87	57.43	68.59
Fedformer	43.31	58.34	69.60
PatchTST	48.17	65.55	73.31
MTand	*52.59*	*70.21*	**83.73**
SeFT	49.26	68.10	79.39

TimelyGPT with time-specific inference achieved the highest recall at $K=5$ and $K=10$, and the second highest at $K=15$, demonstrating its superior performance in long-term forecasting of irregularly-sampled time series

Boldface indicates the highest score among all methods within the same column

**Fig. 5:**
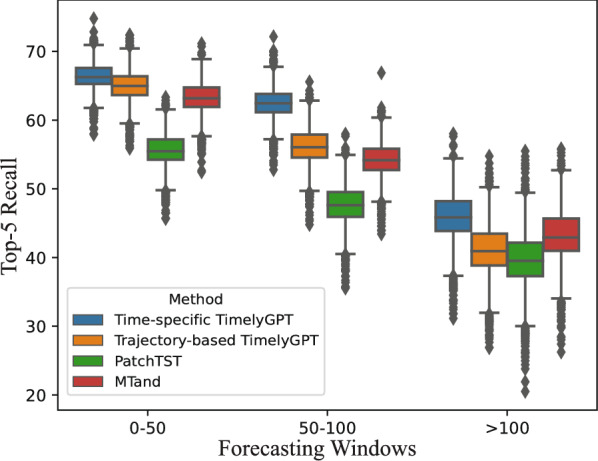
The distribution of top-5 recall performance for TimelyGPT with two inference methods (Time-specific and Trajectory-based), compared to PatchTST and MTand across three forecasting window sizes

**Fig. 6:**
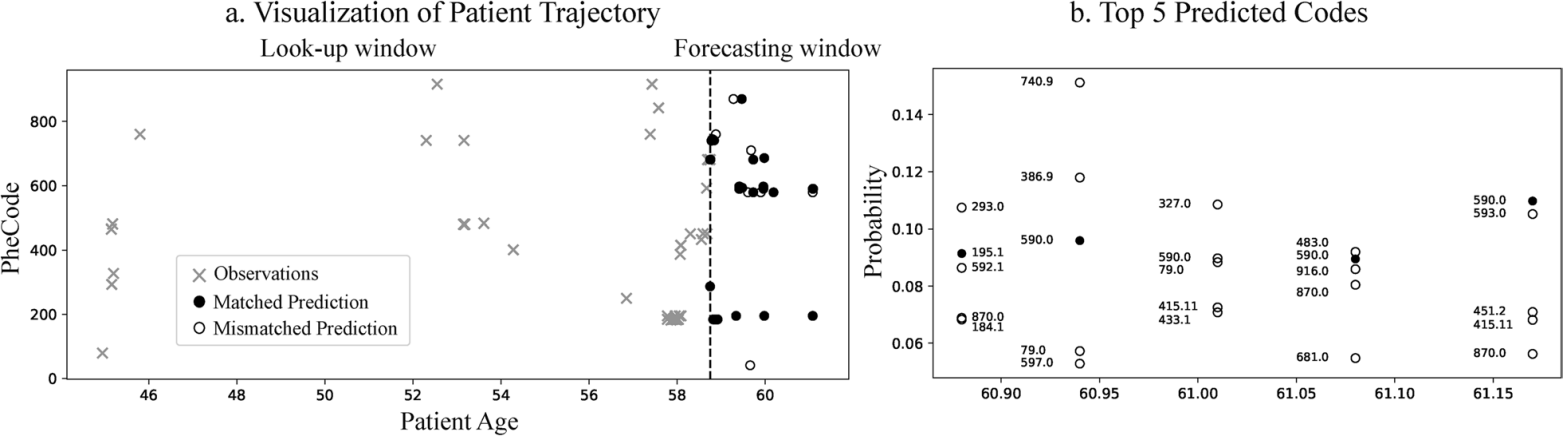
Visualization of a cancer patient’s medical trajectory from the PopHR dataset. **a** Look-up and forecast windows. Matched predictions (solid circles) were identified when the top 5 predicted PheCodes contain the groundtruth. **b** The top 5 predicted PheCodes for the final 5 timesteps of the subject

We visualized the predicted biosignals by TimelyGPT against the leading baselines (PatchTST and DLinear) and the ablated methods (GPT-2 and GPT-2 with RoPE), focusing on sleep stage transitions (Fig. 4). We utilized a 2000-timestep look-up window and a 6000-timestep forecasting window. Forecasting beyond 2000 timesteps is marked as extrapolation, as it exceeds the training length. In the rectal temperature (i.e., trend signal), TimelyGPT ’s forecast aligned well with the groundtruth, effectively capturing distinct trend patterns. Notably, the small bump in the prompt before the 1000-th timestep is a typical indicator for temperature drop. Most models were able to capture it except for DLinear, showing the benefits of pre-training. Beyond the training length of 4000, TimelyGPT demonstrated a clear advantage in accurately extrapolating the rise of the rectal temperature around 7000th timestep while PatchTST and GPT fell behind. The superior extrapolation capabilities of TimelyGPT is attributable to its ability to capture the long-term trends with xPos embedding. In contrast, both PatchTST and vanilla GPT experienced a performance decline, likely due to the dependency on linear mapping as discussed in previous research [34]. Additionally, TimelyGPT exhibits superior extrapolation capabilities over the ablated baseline GPT+RoPE, highlighting its effective trend pattern modeling for extrapolation.

### Forecasting patient diagnosis trajectory

We then applied TimelyGPT and the baseline methods to forecast 315 PheCodes for 489K patients from PopHR. We evaluated the performance using the average top *K* recall at each forecast window. TimelyGPT with time-specific inference outperformed the baselines reaching the highest recall rates of 58.65% and 70.83% at $K=5$ and $K=10$, respectively (Table 2). At $K=15$, TimelyGPT ranked the second-highest recall with 82.69%. In addition, the time-specific inference outperformed the trajectory-based inference, highlighting the advantage of time decay mechanism.

We then examined the distributions of the top-5 recall rates at 3 forecast windows, comparing two inference methods of TimelyGPT with the best transformer baseline PatchTST and the leading irregular time series algorithm MTand (Fig. 5). TimelyGPT ’s time-specific inference consistently outperformed trajectory-based inference as the forecasting window size increases. While both inference methods exhibited similar performance for predicting the first 50 timesteps, time-specific TimelyGPT demonstrated significantly better results beyond 50 timesteps. This improvement is likely due to time-specific inference taking into account the evolving states and the query timestep in the time decay mechanism, enhancing its ability to predict the temporal evolution of healthcare trajectories over irregular intervals. As expected, all models experienced a performance decline in predicting farther future because of the increasing uncertainties. Despite this, TimelyGPT maintained higher and more stable performance within the first 100 steps compared to PatchTST and MTand. Although MTand closely followed to time-specific TimelyGPT for the first 50 timesteps, its performance drastically declined as the forecasting window increases, reflecting its difficulty with extrapolation. These findings highlight the utility of the proposed time-specific inference in leveraging time-decay mechanism to handle irregularly-sampled time series for long-term forecasting.

We visualized the observed and predicted trajectory of a patient with neoplasm and genitourinary diseases (Fig. 6). TimelyGPT with time-specific inference produced a high top-5 recall rate of 85.7% on this patient. Indeed, most of the observed codes were among the top 5 predicted codes by the time-specific TimelyGPT. Zooming into the forecast window (Fig. 6b), TimelyGPT accurately predicted Phecodes 590.0 (Pyelonephritis) three times around the age of 61. TimelyGPT predicted PheCode 740.9 at age 61 with high probability, which appeared twice at ages 52 and 53 in the look-up window. Therefore, TimelyGPT demonstrated a promising direction to forecast patient health state despite the challenges inherent in modeling irregularly-sampled longitudinal EHR data.

**Table 3:** Comparing TimelyGPT with baseline models on the downstream classification and regression tasks using nine biosignal datasets

Task	Classification (Accuracy %)					Regression (MAE)			
Pre-training	Sleep-EDF		PTB-XL			PTB-XL & PPGDalia			
Fine-tuning	Sleep-EDF	Epilepsy	PTB-XL	UKB	EMG	IEEEPPG	RR	HR	SpO2
TimelyGPT	89.21	**92.79**	*86.52*	*80.34*	**95.87**	*26.17*	**2.78**	**8.53**	**4.26**
PatchTST	*89.57*	*91.27*	83.42	**81.36**	*95.23*	**26.08**	*2.89*	9.46	*4.45*
AutoFormer	78.86	84.21	78.51	76.52	88.56	32.18	4.13	13.29	4.95
FedFormer	76.43	81.65	75.53	75.42	85.21	31.11	4.36	13.82	4.75
TimesNet	83.58	85.96	79.38	76.78	89.26	29.95	4.19	13.65	4.83
TST	88.83	88.02	81.86	77.94	94.16	26.81	3.47	12.63	4.95
CRT	**90.12**	91.05	**87.81**	80.18	94.56	26.52	2.96	*9.02*	4.48
TS2Vec	86.21	88.27	82.65	75.32	93.77	27.89	3.53	11.56	4.60
TS-TCC	86.06	89.74	84.66	76.86	93.25	29.32	4.09	13.64	4.86

Bold and italic numbers indicate the best and second best results in each task, respectively

### Classification and regression of biosignals

**Fig. 7:**
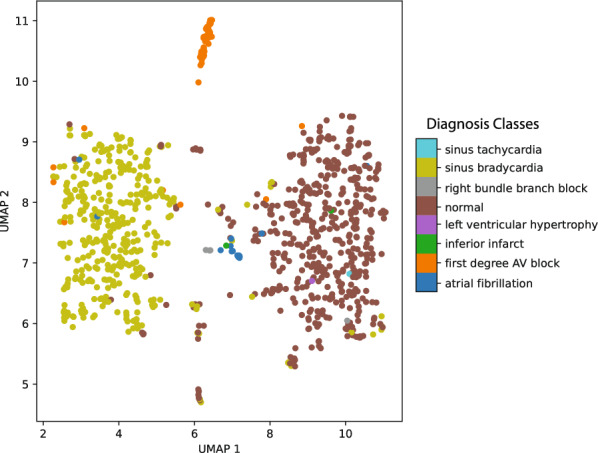
UMAP visualization of TimelyGPT’s learned embeddings for 12-lead ECG sequences from the UK Biobank. Each sequence is colored by eight automated diagnostic classes. The visualization demonstrates distinct clustering of major cardiovascular diagnosis classes

In this section, we aim to demonstrate that TimelyGPT’s next-token prediction pre-training transfers effectively to classification tasks. We pre-trained TimelyGPT on the PTB-XL dataset (21,799 sequences of 12-lead ECGs) and then fine-tuned it for classification tasks using the UK Biobank ECG data (46,321 sequences of 12-lead ECGs). Eight diagnosis labels were extracted from cardiologist text reports using a published rule-based pipeline [55]. To evaluate the learned representations, we applied Uniform Manifold Approximation and Projection (UMAP) technique to 1000 ECG embeddings (Fig. 7) [58]. The visualization reveals distinct clusters for common rhythms, such as sinus bradycardia, first-degree AV block, atrial fibrillation, and normal conditions. It also identified neighboring classes (e.g., atrial fibrillation and right bundle branch block), suggesting that the learned embeddings capture clinically relevant signal patterns.

We further performed quantitative experiment results for the classification and regression tasks, as summarized in Table 3. TimelyGPT achieved the best performance in classifying Epilepsy and EMG and in the regression of RR, HR, and SpO2. Moreover, it ranked second on datasets including PTB-XL, UKB, and IEEEPPG. This superiority highlights the potential of the generative pre-training framework to generalize across datasets [51]. On the other hand, CRT stood out the best when both pre-training and fine-tuning were conducted on the same dataset. Our TimelyGPT outperformed PatchTST for dealing with long sequences, especially in the Sleep-EDF and PTB-XL datasets. This suggests that our proposed convolution-subsampling tokenizer might be more effective at extracting dense information from long sequences. Masking-based transformer PTMs performed poorly due to the distribution shift issue [8]. Models like Autoformer, Fedformer, and TimesNet, which rely on time decomposition and frequency-domain information, were especially affected by distribution shift. Furthermore, consistency-based pretraining methods (PTMs), such as TS2Vec and TS-TCC, generally showed lower performance on these tasks compared to time-series transformer models.

### Disease classification of irregular patient diagnosis sequences

**Fig. 8:**
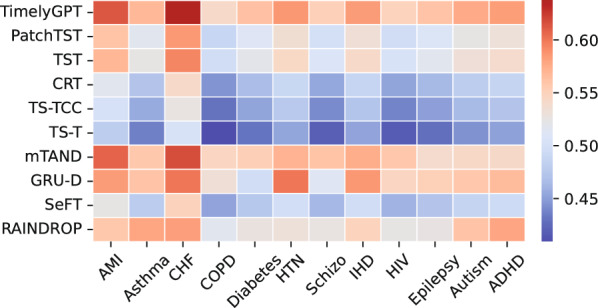
The heatmap displays AUPRC of 9 baselines (rows) and 12 phenotypes (columns) on the PopHR’s irregular-sampled time series. TimelyGPT consistently outperforms other baselines across most phenotypes, especially on AMI, Asthma, and COPD, demonstrating its effectiveness in multi-label classification task

As illustrated in Fig. 8, TimelyGPT outperformed other PTMs by adeptly modeling trend patterns and handling unequal time intervals. This highlights the shortcomings of absolute position embedding, which struggles with phase shifting issue in irregular sequences [59]. TimelyGPT achieved the highest predictive performance in 9 out of 12 phenotypes and ranked second in the remaining 3, demonstrating its effectiveness in classifying multiple phenotype labels from patient sequences. With the aid of time decay, TimelyGPT surpassed other methods specifically designed for irregularly-sampled time series, leading with an average AUPRC of 57.6% and outperforming the next best mTAND (56.4%).

**Table 4:** Ablation results of TimelyGPT w/o specific components, showing forecasting performance for a 6000-timestep window in the Sleep-EDF dataset and top-15 recall rate in the PopHR dataset

Datasets	Sleep-EDF	PopHR
	(6000)	(*K*=5)
TimelyGPT (with Pre-training)	**0.575**	**58.65**
w/o Convolution Subsampling	0.587	–
w/o Temporal Convolution	0.581	57.69
w/o Exponential Decay	0.715	52.50
w/o RoPE (GPT-2)	1.072	50.18
TimelyGPT (w/o Pre-training)	0.641	56.42

Boldface indicates the highest score among all methods within the same column

### Ablation study

To assess the contributions of various components in TimelyGPT, we conducted ablation studies by omitting the key components, including convolution subsampling tokenizer, temporal convolution module, exponential decay, and RoPE relative position embedding. Notably, removing all components results in a vanilla GPT-2. Since exponential decay in xPos depends on RoPE, we cannot assess the impact of exponential decay independently by removing the RoPE component. Additionally, we also ablated the pre-training strategy by training TimelyGPT from scratch on the forecasting tasks. The ablation studies focused on downstream forecasting experiments using the Sleep-EDF and PopHR datasets, corresponding to continuous biosignals and irregularly-sampled time series, respectively. We conducted the ablation on long-term forecasting of 6000 timesteps in the Sleep-EDF dataset and evaluated the top-5 recall scores in the PopHR dataset.

As shown in Table 4, for the Sleep-EDF forecasting task, removing the RoPE component led to the most significant performance degradation (a MAE of 0.357). The removal of exponential decay also led to increase MAE of 0.134, demonstrating its benefits of encoding trend patterns for long-term forecasting. Together, the two ablation experiments show the importance of xPos as our first main contribution (Sect. 3.2). The integration of convolution modules helps TimelyGPT capture local features, although the benefits were smaller compared with other components.

In the forecasting of irregularly-sampled time series, the exponential decay and RoPE components improved performance by 6.15% and 2.32%, respectively. The time decay mechanism encodes trend patterns into the modeling of patients’ health trajectories, making it a promising approach for forecasting irregular clinical diagnoses. Pre-training decreased MAE by 0.066 for forecasting continuous biosignals in Sleep-EDF and increased top K recall rate by 2.21% for forecasting irregularly sampled diagnostic codes.

**Table 5:** Configuration analysis of the Temporal Convolution module on the SleepEDF classification task. DW and PW denote depth-wise and point-wise convolutions, respectively

Configuration	Accuracy (%)
Temporal Convolution (DW + PW)	**89.21**
PW + DW + PW	88.75
DW	88.12
PW	87.56
w/o Temporal Convolution	87.03

Boldface indicates the highest score among all methods within the same column

### Convolution architecture analysis

Combining attention mechanisms with convolution effectively captures global and local dependencies, making it effective for classification tasks [11]. In TimelyGPT, we utilized a depth-wise separable convolution as temporal convolution module, consisting of depth-wise and point-wise convolutions. We compared it with other convolution configurations designed for global–local interactions: (1) a depth-wise convolution only [39]; (2) a combination of point-wise convolution, depth-wise convolution, and another point-wise convolution [11]. We also assessed the efficacy of point-wise convolution. We compare various convolution configurations on the downstream classification task using the SleepEDF dataset, as shown in Table 5. The results indicate that the depth-wise separable convolution in TimelyGPT surpasses other designs in capturing local features and enhancing global–local feature interactions.

## Conclusion

TimelyGPT effectively forecasts long sequence of time-series, utilizing xPos embedding, recurrent attention, and convolution modules. It can accurately extrapolates up to 6000 timesteps given only a 2000-timestep prompt. Moreover, TimelyGPT also effectively forecasts irregularly-sampled time series by conditioning the recurrent Retention on the time. TimelyGPT ’s superiority in classification and regression tasks for both continuous and irregularly-sampled time series is also attributable to its temporal convolution module and time decay mechanism. TimelyGPT ’s superiority in classification and regression tasks for both continuous and irregularly-sampled time series is also attributable to its temporal convolution module and time decay mechanism. This study proposes efficient and effective models for both types of healthcare time-series data, facilitating preventive patient care and early intervention.

One limitation of this study is that TimelyGPT is primarily evaluated on unimodal time-series data, including continuous biosignals and irregularly-sampled EHR data. The real-world clinical data is inherently multi-modal, encompassing electrocardiograms (ECGs), intensive care unit (ICU) measurements, clinical notes, and other heterogeneous sources [60]. A promising direction for future work is to extend TimelyGPT to multi-modal representation learning for more comprehensive patient modeling. Additionally, this study does not explicitly address out-of-domain (OOD) distribution shifts, which commonly occur when transferring pre-trained models across different healthcare populations or institutions. Future work will explore techniques to improve the generalizability of TimelyGPT under such distributional shifts, ensuring its applicability in diverse real-world clinical environments.

## Data Availability

The PopHR data are not available. The eICU data are available under the PhysioNet (https://eicu-crd.mit.edu/). The TimelyGPT software is available from at GitHub: https://github.com/li-lab-mcgill/TimelyGPT

## Appendix 1 Additional details

### Dataset description

*Sleep-EDF dataset for Forecasting. * Sleep-EDF dataset proposed by [40] from PhysioBank [45] contains sleep cassette data obtained from 153 subjects. The collected whole-night polysmnographic (PSG) sleep recordings encompass 7 features: Electroencephalogram (EEG) (from Fpz-Cz and Pz-Oz electrode locations), electrooculogram (EOG) (horizontal), submental chin electromyogram (EMG), and an event marker. EEG and EOG signals were sampled at 100 Hz, while the EMG and event marker were sampled at 1 Hz. The sleep patterns correspond to the PSGs consist of five sleep stages: Wake (W), Non-rapid eye movement (N1, N2, N3) and Rapid Eye Movement (REM). This dataset for ultra-long-term forecasting contains a total of 1.2B timesteps, segmented into 300.7K sequences of 4000 timesteps each.

*Sleep-EDF (Sleep-stage only) dataset for Classification. * We leveraged only the sleep stage of the Sleep-EDF dataset for the classification task following existing studies [2, 61]. In line with conventional practices, we selected the single EEG channel that captures signals from the Fpz-Cz electrode location. Since the Sleep-EDF dataset labels five sleep stages (i.e., W, REM, N1, N2 and N3), we only focused on EEG signals associated with these sleep stages, resulting in a total of 586.4 million timesteps. This dataset was segmented into 195.5K sequences, each with 3000 timesteps.

*Epilepsy Seizure Classification. * Epileptic Seizure Recognition dataset [43] comprises EEG measurements from 500 subjects. This dataset captures brain electrical activity from different regions and states, and are divided into segments of 23.6 s. The original dataset consists of five classes of EEG measuring eyes open, eyes closed, healthy brain region, tumor region, and seizure. The first four classes were merged into a single class as these classes are unrelated to epileptic seizure, enabling a binary classification of epileptic seizures. The dataset for classification consists of 2 M timesteps, divided into 11.5K sequences, each containing 178 timesteps.

*PTB-XL Classification. * Physikalisch Technische Bundesanstalt large scale cardiology database (PTB-XL) [41] from PhysioBank [45] contains 21,837 clinical 12-lead ECG signals (male: 11,379 and female: 10,458) with a duration of 10 s each, sampled with a rate of 500 Hz. These signals are categorized based on a set of twelve leads (I, II, III, AVL, AVR, AVF, V1, V2, V3, V4, V5, V6), where the reference electrodes on the right arm are provided for each signal, resulting in twelve labels for classification. This dataset for classification comprises 21,837 samples, each spanning 5000 timesteps, amounting to a total of 109.2M timesteps.

*UK Biobank ECG Classification. * The UK Biobank is a prospective population cohort comprising approximately 500,000 participants aged 40 to 69 at recruitment [44]. It provides a substantial repository of clinical data, including electrocardiograms (ECGs). In this study, we use 46,321 resting-state clinical 12-lead ECG recordings from the UK Biobank, each comprising 5000 time-steps. Disease labels were derived from ECG machine automated diagnoses using clinically meaningful keywords, such as sinus bradycardia, atrial fibrillation, and normal rhythm, based on criteria from [55]. The selection of cardiovascular disease labels were extracted from cardiologist reports using a pipeline combining n-gram preprocessing, a Lazy Associative Classifier trained on diagnosis text reports, and a rule-based classier for class disambiguation.

*EMG Classification. * EMG dataset from PhysioBank [45] captures the electrical activity resulting from neural stimulation of muscles. This dataset provides insights about muscle functionality and the corresponding nerves responsible for their control. This dataset contains single-channel EMG signals sampled with a rate of 4 KHz. EMG recordings were obtained from the tibialis anterior muscle of volunteers exhibiting different degrees of muscular and neural disorders, resulting in three classification labels. This dataset for classification comprises 306.0K timesteps, segmented into 204 sequences, each consisting of 1500 timesteps.

*PPG-Dalia Regression. * PPG-Dalia dataset [5] is available from Monash University, UEA&UCR Time Series Regression Archive [46]. This dataset captures photoplethysmograph (PPG) data for motion compensation and heart rate estimation. Data was collected from 15 subjects engaging in daily life activities using both wrist-worn (Empatica E4) and chest-worn (RespiBAN Professional) devices. All signals were sampled at 700 Hz. The ECG recording serve as a ground truth for heart rate. The PPG and 3D-accelerometer data are used to estimate heart rate, while accounting for motion artefacts. This dataset with a total of 16.6M timesteps was divided into 64,697 sequences, with varied lengths of 256 or 512 timesteps in different dimensions. The pre-training on this PPG-Dalia as well as the PTB-XL dataset was utilized for fine-tuning regression.

*IEEEPPG Regression. * IEEEPPG dataset [56] is sourced from Monash University, UEA&UCR Time Series Regression Archive [46]. This dataset aims to estimate heart rate by utilizing PPG and ECG signals. The dataset includes two-channel PPG signals, three-axis acceleration signals, and one-channel ECG signals. These signals were recorded simultaneously and sampled at a rate of 125 Hz. This dataset for regression contains 3.1M timesteps, segmented into 3.096K sequences of 1000 timesteps each.

*RR, HR and SpO2 Regression. * BIDMC Respiratory Rate (RR), heart rate (HR) and blood oxygen saturation level (SpO2) datasets [62] are available at Monash University, UEA&UCR Time Series Regression Archive [46]. These datasets were obtained from the Physionet’s BIDMC PPG and Respiration dataset [45]. They contain PPG and ECG signals sampled at a rate of 125 Hz, which are designed for estimating RR, HR, and SpO2. The three datasets for regression were segmented into sequences of 4000 timesteps each, yielding: (1) 7,870 sequences in the RR dataset, totaling 31.5M timesteps; (2) 7949 sequences in the HR dataset, encompassing 31.8M timesteps; (1) 7949 sequences in the SpO2 dataset, also amounting to 31.8M timesteps.

*PopHR database. * The database hosts a massive amount of longitudinal heterogeneous claim data from the provincial government health insurer in Quebec, Canada (Régie de l’assurance maladie du Québec, RAMQ) on health service use [47, 48]. In total, there are approximately 1.3 million participants in the PopHR database, which represents a randomly sampled 25% of the population in the metropolitan area of Montreal between 1998 and 2014. Cohort membership is maintained dynamically by removing deceased residents and actively enrolling newborns and immigrants.

### Data pre-processing

For forecasting experiment, we utilized the entire seven-variates Sleep-EDF dataset, excluding the instances with unknown sleep stages. We divided the dataset into training (80%), validation (10%) and testing (10%) sets. We used the whole train set for pre-training and 20% of train set for fine-tuning. During pre-training, TimelyGPT is pre-trained on non-overlapped sequences with 4000 timesteps. To use Sleep-EDF dataset for classification, we only utilized the single EEG (Fpz-Cz electrode signal) channel and considered sleep stage from the Sleep-EDF dataset. All datasets utilized for classification and regression tasks are divided into training (85%), validation (5%), and testing (10%) sets.

For the irregularly-sampled time series on the PopHR database, we converted ICD-9 codes to PheCodes using the expert-defined PheWAS catalog [49, 50]. We calculated the prevalence ratio for each PheCode in the true patient population and the general population for 12 disease labels. For each disease label, we chose PheCodes that either had a prevalence ratio exceeding 5 or were among the top 5 most prevalent. Furthermore, we excluded patients with fewer than 10 occurrences, resulting in a processed dataset of 47,000 patients and 2.2 million records. Due to data sparsity, patients with very few PheCodes (i.e., short sequences) often lack sufficient clinical context for learning meaningful patterns and making accurate classification. To address it, we empirically tested several thresholds (i.e., 5, 10, 15) for the minimum number of PheCodes per patient. A threshold of 10 provides a good balance between maintaining a large enough dataset and achieving strong classification performance. The processed dataset is then divided into training (80%), validation (10%), and testing (10%) sets. In total, 133 unique PheCodes were included in our experiment. Given that diagnoses are discrete value, there was no need to utilize the convolution-subsampling tokenizer. Furthermore, we specified a patch size of 2 for PatchTST.

### Additionally experimental information

We summarize the setup of model architecture for TimelyGPT and other baselines in Table 6, 7, 8, 9.

**Table 6:** The statistics of datasets

Dataset	Task	Features	Timesteps	Sequences	Length	Classes
Sleep-EDF	Forecasting	7	1.2B	400.7K	3000	None
Sleep-EDF (sleep)	Classification	1	586.4M	195.5K	3000	5
Epilepsy	Classification	1	2.0M	11.5K	178	2
PTB-XL	Classification	12	109.2M	21,837	5000	5
UK Biobank	Classification	12	231.6M	46,312	5000	8
EMG	Classification	1	306.0K	204	1,500	3
PPG-Dalia	Regression	4	16.6M	64,697	256 & 512$^*$	None
IEEEPPG	Regression	5	3.1M	3,096	1000	None
RR	Regression	2	31.5M	7,870	4000	None
HR	Regression	2	31.8M	7,949	4000	None
SpO2	Regression	2	31.8M	7,949	4000	None

Note that the sequence length of the PPG-Dalia dataset varies across different dimensions

**Table 7:** Configurations of TimelyGPT, transformer baselines, and recurrent models across different datasets

	Sleep-EDF	PTB-XL (PPGDalia)	PopHR
Data Size (timesteps)	1.2B	109.2M (6.6M)	54.9M
Model Parameters	18M	7.5M	6M
TimelyGPT			
Decoder Layers	12	8	8
Heads	8	8	4
Dim (${{\varvec{Q}}}$, ${{\varvec{K}}}$, ${{\varvec{V}}}$, FF)	320,320,640,640	240,240,480,480	200,200,400,400
Transformer baselines including Encoder-decoder and Encoder-only models			
Enc-Dec Layers	6 & 6	4 & 4	4 & 4
Encoder Layers	12	8	8
Decoder Layers	12	8	8
Heads	8	8	4
Dim (${{\varvec{Q}}}$, ${{\varvec{K}}}$, ${{\varvec{V}}}$, FF)	384,384,384,1536	288,288,288,1152	200,200,200,400
Recurrent Models			
Layers	12	8	8
Dim	384	288	200

**Table 8:** Pre-training time per epoch (in hours) for TimelyGPT and baselines on the SleepEDF and PopHR datasets, measured using an NVIDIA A100 GPU (40GB)

Method/dataset (h)	SleepEDF	PopHR
Data size (timesteps)	1.2B	54.9M
TimelyGPT	**1.4**	**0.6**
GPT-2	4.2	1.4
PatchTST	5.8	1.7
Informer	3.8	1.3
Fedformer	3.5	2.3
Autoformer	4.5	2.1

Boldface indicates the highest score among all methods within the same column

**Table 9:** Forecasting performance of TimelyGPT on patient sequences with varying record densities using the PopHR dataset

Record density (%)	TimelyGPT Recall@10 (%)	Last Visit Recall@10 (%)
$< 25$	58.2	22.1
$25 - 50$	63.2	24.3
$50 - 75$	72.4	26.8
$> 75$	77.1	25.4

Record density is defined as the proportion of observed records within the look-up window. We report Recall@10 for both full-sequence forecasting and a baseline using only the last visit (a random prediction)

## References

[1] Ma Q, Liu Z, Zheng Z, Huang Z, Zhu S, Yu Z, et al. A survey on time-series pre-trained models. IEEE Trans Knowl Data Eng. 2024. 10.1109/TKDE.2024.3475809.

[2] Eldele E, Ragab M, Chen Z, Wu M, Kwoh CK, Li X, Guan C. Time-series representation learning via temporal and contextual contrasting. Proceedings of the Thirtieth International Joint Conference on Artificial Intelligence, IJCAI-21, pp. 2352–2359 (2021)

[3] Stirling R, Cook M, Grayden D, Karoly P. Seizure forecasting and cyclic control of seizures. Epilepsia. 2020. 10.1111/epi.16541. 10.1111/epi.16541 32712968

[4] Phan H, Chen OY, Tran MC, Koch P, Mertins A, Vos MD. XSleepNet: multi-view sequential model for automatic sleep staging. IEEE Trans Pattern Anal Mach Intell. 2021. 10.1109/tpami.2021.3070057. 10.1109/TPAMI.2021.3070057 33788679

[5] Reiss A, Indlekofer I, Schmidt P, Van Laerhoven K. Deep PPG: large-scale heart rate estimation with convolutional neural networks. Sensors. 2019. 10.3390/s19143079. 10.3390/s19143079 PMC6679242 31336894

[6] Zhang X, Zeman M, Tsiligkaridis T, Zitnik M. Graph-guided network for irregularly sampled multivariate time series. International Conference on Learning Representations, ICLR (2022)

[7] Zerveas G, Jayaraman S, Patel D, Bhamidipaty A, Eickhoff C. A transformer-based framework for multivariate time series representation learning. KDD ’21 (2021). 10.1145/3447548.3467401.

[8] Zhang W, Yang L, Geng S, Hong S. Self-supervised time series representation learning via cross reconstruction transformer. IEEE Trans Neural Netw Learn Syst. 2023. 10.1109/TNNLS.2023.3292066. 10.1109/TNNLS.2023.3292066 37478042

[9] Jiang J, Han C, Zhao WX, Wang J. Pdformer: propagation delay-aware dynamic long-range transformer for traffic flow prediction. Proc AAAI Conf Artif Intell. 2023;37:4365–73.

[10] Padhi I, Schiff Y, Melnyk I, Rigotti M, Mroueh Y, Dognin P, Ross J, Nair R, Altman E. Tabular transformers for modeling multivariate time series. ICASSP 2021-2021 IEEE International Conference on Acoustics, Speech and Signal Processing (ICASSP), pp. 3565–3569 (2021). IEEE

[11] Gulati A, Qin J, Chiu CC, Parmar N, Zhang Y, Yu J, Han W, Wang S, Zhang Z, Wu Y, Pang R. Conformer: Convolution-augmented transformer for speech recognition, 5036–5040 (2020). 10.21437/Interspeech.2020-3015

[12] Zhou H, Zhang S, Peng J, Zhang S, Li J, Xiong H, et al. Informer: beyond efficient transformer for long sequence time-series forecasting. Proc AAAI Conf Artif Intell. 2021;35:11106–15. 10.1609/aaai.v35i12.17325.

[13] Wu H, Xu J, Wang J, Long M. Autoformer: decomposition transformers with auto-correlation for long-term series forecasting. Adv Neural Inf Process Syst (2021)

[14] Zhou T, Ma Z, Wen Q, Wang X, Sun L, Jin R. FEDformer: Frequency enhanced decomposed transformer for long-term series forecasting. Proc. 39th International Conference on Machine Learning (ICML 2022) (2022)

[15] Kaplan J, McCandlish S, Henighan T, Brown TB, Chess B, Child R, Gray S, Radford A, Wu J, Amodei D. Scaling Laws for Neural Language Models (2020)

[16] Zeng A, Chen M, Zhang L, Xu Q. Are transformers effective for time series forecasting? Proc AAAI Conf Artif Intell. 2023;37:11121–8. 10.1609/aaai.v37i9.26317.

[17] Yue Z, Wang Y, Duan J, Yang T, Huang C, Tong Y, et al. Ts2vec: towards universal representation of time series. Proc AAAI Conf Artif Intell. 2022;36:8980–7. 10.1609/aaai.v36i8.20881.

[18] Tang W, Long G, Liu L, Zhou T, Blumenstein M, Jiang J. Omni-scale CNNs: a simple and effective kernel size configuration for time series classification. Int Conf Learn Represent (2021)

[19] Zhai X, Kolesnikov A, Houlsby N, Beyer L. Scaling vision transformers. In: Proceedings of the IEEE/CVF Conference on Computer Vision and Pattern Recognition (CVPR), pp. 12104–12113 (2022)

[20] Sun Y, Dong L, Patra B, Ma S, Huang S, Benhaim A, Chaudhary V, Song X, Wei F. A length-extrapolatable transformer. In: Rogers, A., Boyd-Graber, J., Okazaki, N. (eds.) Proceedings of the 61st Annual Meeting of the Association for Computational Linguistics (Volume 1: Long Papers), pp. 14590–14604. Association for Computational Linguistics, Toronto, Canada (2023). 10.18653/v1/2023.acl-long.816 . https://aclanthology.org/2023.acl-long.816

[21] Sun Y, Dong L, Huang S, Ma S, Xia Y, Xue J, Wang J, Wei F. Retentive network: A successor to transformer for large language models (2023)

[22] Vaswani A, Shazeer N, Parmar N, Uszkoreit J, Jones L, Gomez AN, Kaiser L, Polosukhin I. Attention is all you need. In: Proceedings of the 31st International Conference on Neural Information Processing Systems. NIPS’17, pp. 6000–6010. Curran Associates Inc., Red Hook (2017)

[23] Tolstikhin I, Houlsby N, Kolesnikov A, Beyer L, Zhai X, Unterthiner T, Yung J, Steiner A, Keysers D, Uszkoreit J, Lucic M, Dosovitskiy A. Mlp-mixer: an all-mlp architecture for vision. In: Proceedings of the 35th International Conference on Neural Information Processing Systems. NIPS ’21. Curran Associates Inc., Red Hook (2024)

[24] Poli M, Massaroli S, Nguyen E, Fu DY, Dao T, Baccus S, Bengio Y, Ermon S, Ré C. Hyena hierarchy: towards larger convolutional language models. In: Proceedings of the 40th International Conference on Machine Learning. ICML’23 (2023)

[25] Peng B, Alcaide E, Anthony Q, Albalak A, Arcadinho S, Cao H, Cheng X, Chung M, Grella M, GV KK, He X, Hou H, Kazienko P, Kocon J, Kong J, Koptyra B, Lau H, Mantri KSI, Mom F, Saito A, Tang X, Wang B, Wind JS, Wozniak S, Zhang R, Zhang Z, Zhao Q, Zhou P, Zhu J, Zhu RJ. RWKV: Reinventing RNNs for the Transformer Era (2023)

[26] Katharopoulos A, Vyas A, Pappas N, Fleuret F. Transformers are rnns: fast autoregressive transformers with linear attention. Proceedings of the 37th International Conference on Machine Learning. ICML’20 (2020)

[27] Gu A, Goel K, Ré C. Efficiently modeling long sequences with structured state spaces. In: The International Conference on Learning Representations (ICLR) (2022)

[28] Shaw P, Uszkoreit J, Vaswani A. Self-attention with relative position representations. In: Proceedings of the 2018 Conference of the North American Chapter of the Association for Computational Linguistics: Human Language Technologies, NAACL-HLT, New Orleans, Louisiana, USA, June 1-6, 2018, Volume 2 (Short Papers), pp. 464–468 (2018). 10.18653/V1/N18-2074

[29] Touvron H, Lavril T, Izacard G, Martinet X, Lachaux M-A, Lacroix T, et al. LLaMA: open and efficient foundation language models (2023).

[30] Penedo G, Malartic Q, Hesslow D, Cojocaru R, Cappelli A, Alobeidli H, et al. The RefinedWeb dataset for Falcon LLM: outperforming curated corpora with web data, and web data only (2023).

[31] Su J, Ahmed M, Lu Y, Pan S, Bo W, Liu Y. Roformer: enhanced transformer with rotary position embedding. Neurocomput. 2024. 10.1016/j.neucom.2023.127063.

[32] Press O, Smith N, Lewis M. Train short, test long: attention with linear biases enables input length extrapolation. In: International Conference on Learning Representations (2022)

[33] Nie Y, Nguyen H, Sinthong P, Kalagnanam J. A time series is worth 64 words: long-term forecasting with transformers. Int Conf Learn Representations (2023)

[34] Li Z, Qi S, Li Y, Xu Z. Revisiting long-term time series forecasting: an investigation on linear mapping (2023)

[35] Ahuja Y, Zou Y, Verma A, Buckeridge D, Li Y. Mixehr-guided: a guided multi-modal topic modeling approach for large-scale automatic phenotyping using the electronic health record. J Biomed Inform. 2022;134:104190. 36058522 10.1016/j.jbi.2022.104190

[36] Song Z, Hu Y, Verma A, Buckeridge DL, Li Y. Automatic phenotyping by a seed-guided topic model. Proceedings of the 28th ACM SIGKDD Conference on Knowledge Discovery and Data Mining. KDD ’22 (2022). 10.1145/3534678.3542675 .

[37] LeCun Y, Bengio Y. Convolutional networks for images, speech, and time series, pp. 255–258. MIT Press, Cambridge (1998)

[38] Chollet F. Xception: Deep learning with depthwise separable convolutions. 2017 IEEE Conference on Computer Vision and Pattern Recognition (CVPR), 1800–1807 (2016)

[39] Wu* Z, Liu* Z, Lin J, Lin Y, Han S. Lite transformer with long-short range attention. In: International Conference on Learning Representations (2020)

[40] Kemp B, Zwinderman AH, Tuk B, Kamphuisen HAC, Oberye JJL. Analysis of a sleep-dependent neuronal feedback loop: the slow-wave microcontinuity of the EEG. IEEE Trans Biomed Eng. 2000;47(9):1185–94. 10.1109/10.867928. 11008419 10.1109/10.867928

[41] Alday EAP, Gu A, Shah AJ, Robichaux C, Wong A-KI, Liu C, et al. Classification of 12-lead ECGs: the physionet/computing in cardiology challenge. Physiol Meas. 2020;41:124003. 10.1088/1361-6579/abc960. 10.1088/1361-6579/abc960 PMC8015789 33176294

[42] Reiss A, Indlekofer I, Schmidt P, Laerhovenn KV. Deep PPG: large-scale heart rate estimation with convolutional neural networks. Sensors. 2019;19(14):3079. 10.3390/s19143079. 31336894 10.3390/s19143079 PMC6679242

[43] Andrzejak RG, Lehnertz K, Mormann F, Rieke C, David P, Elger CE. Indications of nonlinear deterministic and finite-dimensional structures in time series of brain electrical activity: dependence on recording region and brain state. Phys Rev E. 2001;6:061907. 10.1103/PhysRevE.64.061907 11736210

[44] Bycroft C, Freeman C, Petkova D, Band G, Elliott LT, Sharp K, et al. The UK biobank resource with deep phenotyping and genomic data. Nature. 2018;562(7726):203–9. 10.1038/s41586-018-0579-z. 30305743 10.1038/s41586-018-0579-z PMC6786975

[45] Goldberger AL, Amaral L, Glass L, Hausdorff J, Ivanov PC, Mark R, et al. Physiobank, physiotoolkit, and physionet: components of a new research resource for complex physiologic signals. Circulation. 2000;101(23):215–20. 10.1161/01.cir.101.23.e215. 10.1161/01.cir.101.23.e215 10851218

[46] Tan CW, Bergmeir C, Petitjean F, Webb GI. Time series extrinsic regression. Data Min Knowl Disc. 2021. 10.1007/s10618-021-00745-9. 10.1007/s10618-021-00745-9 PMC7951134 33727888

[47] Shaban-Nejad A, Lavigne M, Okhmatovskaia A, Buckeridge D. Pophr: a knowledge-based platform to support integration, analysis, and visualization of population health data: the population health record (pophr). Ann N Y Acad Sci. 2016;1387(1):44–53. 10.1111/nyas.13271. 27750378 10.1111/nyas.13271

[48] Yuan M, Powell G, Lavigne M, Okhmatovskaia A, Buckeridge D. Initial usability evaluation of a knowledge-based population health information system: yhe population health record (pophr). AMIA—Annual Symposium proceedings. AMIA Symposium 2017, pp. 1878–1884 (2018) PMC5977587 29854259

[49] Denny J, Bastarache L, Ritchie M, Carroll R, Zink R, Mosley J, et al. Systematic comparison of phenome-wide association study of electronic medical record data and genome-wide association study data. Nat Biotechnol. 2013;31(12):1102–11. 10.1038/nbt.2749. 24270849 10.1038/nbt.2749 PMC3969265

[50] Denny JC, Ritchie MD, Basford MA, Pulley JM, Bastarache L, Brown-Gentry K, et al. PheWAS: demonstrating the feasibility of a phenome-wide scan to discover gene–disease associations. Bioinformatics. 2010;26(9):1205–10. 10.1093/bioinformatics/btq126. 20335276 10.1093/bioinformatics/btq126 PMC2859132

[51] Radford A, Wu J, Child R, Luan D, Amodei D, Sutskever I. Language models are unsupervised multitask learners. (2019). https://api.semanticscholar.org/CorpusID:160025533

[52] Wu H, Hu T, Liu Y, Zhou H, Wang J, Long M. Timesnet: temporal 2D-variation modeling for general time series analysis. International Conference on Learning Representations (2023)

[53] Shukla SN, Marlin B. Multi-time attention networks for irregularly sampled time series. In: International Conference on Learning Representations (2021) PMC12927590 41737905

[54] Horn M, Moor M, Bock C, Rieck B, Borgwardt K. Set functions for time series. In: III, H.D., Singh, A. (eds.) Proceedings of the 37th International Conference on Machine Learning. Proceedings of Machine Learning Research. 119:4353–4363 (2020)

[55] Ribeiro AH, Ribeiro MH, Paixão GMM, Oliveira DM, Gomes PR, Canazart JA, et al. Automatic diagnosis of the 12-lead ECG using a deep neural network. Nat Commun. 2020;11(1):1760. 10.1038/s41467-020-15432-4. 32273514 10.1038/s41467-020-15432-4 PMC7145824

[56] Zhang Z, Pi Z, Liu B. Troika: a general framework for heart rate monitoring using wrist-type photoplethysmographic signals during intensive physical exercise. IEEE Trans Biomed Eng. 2015;62(2):522–31. 10.1109/TBME.2014.2359372. 25252274 10.1109/TBME.2014.2359372

[57] Che Z, Purushotham S, Cho K, Sontag D, Liu Y. Recurrent neural networks for multivariate time series with missing values. Sci Rep. 2018;8(1):6085. 10.1038/s41598-018-24271-9. 29666385 10.1038/s41598-018-24271-9 PMC5904216

[58] McInnes L, Healy J, Saul N, Grossberger L. UMAP: uniform manifold approximation and projection. J Open Source Softw. 2018;3(29):861.

[59] Sinha K, Kazemnejad A, Reddy S, Pineau J, Hupkes D, Williams A. The curious case of absolute position embeddings (2022)

[60] Johnson AEW, Bulgarelli L, Shen L, Gayles A, Shammout A, Horng S, et al. Mimic-iv, a freely accessible electronic health record dataset. Sci Data. 2023;10:1. 36596836 10.1038/s41597-022-01899-x PMC9810617

[61] Zhang X, Zhao Z, Tsiligkaridis T, Zitnik M. Self-supervised contrastive pre-training for time series via time-frequency consistency. Proceedings of Neural Information Processing Systems, NeurIPS (2022)

[62] Pimentel MAF, Johnson AEW, Charlton PH, Birrenkott D, Watkinson PJ, Tarassenko L, et al. Toward a robust estimation of respiratory rate from pulse oximeters. IEEE Trans Biomed Eng. 2017;64(8):1914–23. 10.1109/TBME.2016.2613124. 27875128 10.1109/TBME.2016.2613124 PMC6051482

